# Innate-Immunity Genes in Obesity

**DOI:** 10.3390/jpm11111201

**Published:** 2021-11-14

**Authors:** Svetlana V. Mikhailova, Dinara E. Ivanoshchuk

**Affiliations:** 1 Federal Research Center, Institute of Cytology and Genetics, Siberian Branch of Russian Academy of Sciences (SB RAS), 630090 Novosibirsk, Russia; dinara2084@mail.ru; 2 Institute of Internal and Preventive Medicine—Branch of Institute of Cytology and Genetics, SB RAS, 630004 Novosibirsk, Russia

**Keywords:** obesity, adipose tissue, innate immunity, gene knockout, polymorphism, genetic predisposition, microbiome

## Abstract

The main functions of adipose tissue are thought to be storage and mobilization of the body’s energy reserves, active and passive thermoregulation, participation in the spatial organization of internal organs, protection of the body from lipotoxicity, and ectopic lipid deposition. After the discovery of adipokines, the endocrine function was added to the above list, and after the identification of crosstalk between adipocytes and immune cells, an immune function was suggested. Nonetheless, it turned out that the mechanisms underlying mutual regulatory relations of adipocytes, preadipocytes, immune cells, and their microenvironment are complex and redundant at many levels. One possible way to elucidate the picture of adipose-tissue regulation is to determine genetic variants correlating with obesity. In this review, we examine various aspects of adipose-tissue involvement in innate immune responses as well as variants of immune-response genes associated with obesity.

## 1. Adipose-Tissue Types and Functions

Adipose tissue normally consists of adipocytes (fewer than 15% of cells by number and more than 90% by volume), preadipocytes (~50% of cells), fibroblasts, endothelial cells, and immune cells (macrophages, dendritic cells (DCs), T and B cells, natural killer (NK) cells, and eosinophils) [1,2]. Depending on the type (white, beige, brown, or pink), adipocytes contain lipid droplets of different sizes and different numbers of mitochondria and also differ in the gene expression profile, their origin, and function in the body [1,3,4,5]. In an adult, the overwhelming majority of adipocytes are white. They differentiate from preadipocytes, which in turn are derived from mesenchymal stem cells. The size of white adipocytes can vary between 20 and 200 µm depending on the size of the unilocular lipid droplet inside. The droplet has a hydrophobic core of neutral lipids surrounded by a phospholipid monolayer. White adipose tissue (WAT) is usually categorized into two main depots: visceral (including perirenal, gonadal, epicardial, retroperitoneal, omental, and mesenteric) and subcutaneous (normally ~80% in humans). The percentage of subcutaneous fat is on average higher in women than in men [6]. Some researchers also distinguish a bone marrow fat depot. White adipocytes in various depots have dissimilar origins because they differ in developmental gene expression [7]. There are differences in the expression of cytokines CCL2, CCL5, CCL20, colony-stimulating factor 1, interleukin (IL)- 1β, IL-6, IL-8, tumor necrosis factor (TNF), PAI-1, leptin, and pattern recognition receptors (PRRs) between visceral and subcutaneous WAT in health and disease [8,9,10,11]. In addition, there are dissimilarities among the populations of white adipocytes within the same depot; this observation may explain metabolic differences among WAT depots [12]. Brown adipocytes are highly active metabolically and are necessary for thermoregulation; their number decreases with age, and they are located in specialized depots in cervical, supraclavicular, and paravertebral regions of the human body [13]. Beige adipocytes are inducible thermogenic cells and contain a multilocular lipid droplet just as brown adipocytes do but are located within WAT [14]. Pink adipocytes are reported to be present in mammary glands and to be involved in post-pregnancy lactation. Their transdifferentiation from white adipocytes has been shown [15].

In live humans, there is periodic renewal of adipocytes: preadipocytes differentiate into mature adipocytes, and researchers have described processes of “browning”, “whitening”, and “pinking” of white, pink, and brown fat in response to changing external conditions through either transdifferentiation or de novo differentiation from adipocyte progenitors [5,14,15]. Elimination of large adipocytes is a major challenge for species with a long lifespan. The safest way to utilize dead cells is apoptosis, i.e., programmed cell death preventing inflammation. During apoptosis, a dying cell undergoes nuclear fragmentation, chromatin condensation, and in a final stage, splits into apoptotic bodies, which are quickly engulfed by macrophages. Large lipid droplets, which make up the bulk of a large adipocyte, on the one hand, reduce the ratio of the cell surface to its volume, thereby hampering classic apoptosis with membrane blebbing and the formation of apoptotic bodies. On the other hand, free fatty acids, especially their oxidized derivatives (whose amount is greater in senescent adipocytes), entering the intercellular space during the destruction of adipocyte membranes can be recognized by several types of PRRs, including Toll-like receptors (TLRs), NOD-like receptors (NLRs), and free-fatty-acid receptors as damage-associated molecular patterns (DAMPs) and induce an inflammatory response [16].

Therefore, adipose tissue requires a finely tuned mechanism to control the rate of destruction of senescent cells, which allows the restraint of possible acute immune reactions. This is partially achieved via control over the total number of adipocytes in the body; it has been shown that, normally in an adult, a change in the volume of adipose tissue is mostly independent from alterations of the number of cells and is determined by a change in their size [17]. Up to 10% of adipocytes in both subcutaneous and visceral adipose tissues are annually cleaned up by macrophages [18]. Apoptosis of adipocytes during lipodystrophy progression has been documented [19]. By contrast, another type of cell death appears to be common in normal adipose tissue: trogocytosis [20,21]. The latter is the biting off of small portions of an adipocyte, including the content of the lipid droplet, by macrophages; this process enables controlled destruction of a large cell without the release of fatty acids into the extracellular space to prevent a lipotoxic effect. It has been demonstrated that during trogocytosis, macrophages produce a large amount of proinflammatory IL-6 but not TNF or IL-1β [20].

## 2. Adipose Tissue in Obesity

Obesity (body-mass index (BMI) > 30) is the most common problem linked with adipose-tissue dysfunction in humans and is a risk factor for type 2 diabetes mellitus, nonalcoholic fatty liver disease, atherosclerosis, hypertension, dyslipidemia, coagulopathies, certain types of cancer, and autoimmune diseases. Obesity is characterized by a significant increase in the size of white adipocytes. It is reported that the highest risk of obesity complications is posed by the growth of abdominal WAT. In obese patients, omental adipocytes are 15–30% smaller than abdominal subcutaneous adipocytes, but there is evidence that this is true only for women [22,23]. Sprouting of blood vessels and remodeling of the extracellular matrix, including collagen deposition, are observed in obese WAT; adipocyte death and preadipocyte differentiation are enhanced, and the turnover is accelerated 2–30-fold at the whole-body level [17,18,24]. In obesity, adipocyte death is described as either necrosis-like death [24] or pyroptosis [25]. Macrophages fuse to form giant multinucleated structures, containing up to 10% of macrophage-associated nuclei in adipose tissue. They seem to specialize in large-adipocyte destruction, and up to 15 macrophages aggregate around each dead adipocyte creating a “crownlike” structure [24,26]. For this purpose, additional monocytes and macrophages are attracted to senescent adipocytes. Pyroptosis is an inflammatory type of programmed cell death that is initiated by the engagement of intracellular NLRs and leads to disruption of the plasma membrane and a release of proinflammatory cytokines IL-1β and IL-18 into the extracellular space. In contrast to apoptosis, chromatin condensation and apoptotic-body formation do not occur. Massive migration of macrophages, neutrophils, mast cells, and T and B cells into fat depots alters the immune status of adipose tissue, and obesity is described as low-grade chronic inflammation of WAT. This inflammation is characterized by an elevated level of proinflammatory cytokines and C-reactive protein (CRP) and lower levels of anti-inflammatory cytokines [9,27,28]. In morbidly obese persons, up to 65% of the stromal-vascular cells of visceral WAT are leukocytes. Adipocyte size correlates with the number of macrophages within each fat depot [22,29]. In humans, macrophage counts are 2- to 2.5-fold higher in omental fat than in subcutaneous fat [9,22]. In the morbidly obese, subcutaneous fat contains fewer macrophages in “crowns” than omental fat does [22]. Visceral fat and subcutaneous fat produce different amounts of proinflammatory cytokines [9,30]. Larger adipocytes correlate with a more proinflammatory adipokine profile (including TNF, leptin, resistin, visfatin, IL-6, and adiponectin) [31]. In obesity, aside from an increase in the number of immune cells, their status changes. The proinflammatory “classically activated” M1 macrophages and anti-inflammatory M2 macrophages possess completely different gene expression patterns. M1 macrophages produce cytokines TNF, IL-6, IL-1β, IL-12, and IL-23 to enhance inflammation, whereas M2 macrophages produce cytokines IL-4, IL-13, and TGF-β to resolve it. In obesity, there is a switch from the M2 to M1 macrophage phenotype and an alteration of the M2:M1 ratio [32,33]. As a result, an inflammatory milieu is formed in abdominal WAT and is believed to provoke diseases related to abdominal obesity.

There is no consensus on what triggers adipose-tissue inflammation in obesity. In mice, adipocyte hypertrophy, rather than overall obesity, is the major contributor to adipose-tissue inflammation [34]. It is assumed that this inflammation is caused by the necrosis of adipocytes owing to their hypertrophy and impaired interaction with the extracellular matrix [35,36]. Nonetheless, despite the large adipocyte size in subcutaneous fat, concentrations of proinflammatory cytokines and the number of recruited macrophages are lower there [30]. WAT hypoxia is regarded as another trigger of inflammation in obesity and develops due to an insufficient blood supply after the tissue growth [37]. Another hypothesis involves greater intestinal permeability for lipopolysaccharides (LPSs) produced by intestinal Gram-negative bacteria in people with higher nutritional energy intake, which results in higher levels of circulating LPSs [38,39]. Gut-derived LPSs can initiate an inflammatory cascade through the triggering of PRRs, such as TLRs on the surface of macrophages and adipocytes [40]. It is suggested that intestinal LPSs may be an important trigger of inflammation, especially in mesenteric fat [41], but most likely merely enhance the effects of an earlier trigger of inflammation. Disruption of energy homeostasis as a whole, which drives the activation of stress-induced kinases and their downstream signaling cascades, is also thought to be a trigger [42]. Investigators have identified DAMPs whose concentration changes in adipose tissue before adipocyte hypertrophy and macrophage accumulation and hence before the development of hypoxia and mechanical stress. One of the alarmins, S100A8, is detectable in fat tissue after 5 days of a high-fat high-sugar diet [43]. There is a theory that low-grade inflammation is an adaptive response to obesity and is aimed at enhancing the differentiation of preadipocytes, remodeling of the extracellular matrix, and angiogenesis [4,27,42,44,45].

It has been hypothesized that obesity-induced insulin resistance precedes macrophage accumulation and inflammation in adipose tissue, not vice versa. It has been shown that genetically induced adipose-tissue–specific insulin resistance in mice causes the production of a chemoattractant protein called MCP1, which recruits monocytes and activates proinflammatory macrophages [46].

## 3. Innate-Immunity Genes in Obesity

Approximately 40–70% of interindividual variance of the BMI has been attributed to genetic factors [47,48]. Several genes are known to be associated with monogenic types of obesity (e.g., *LEP*, *LEPR*, *MC4R*, and *POMC*); however, most cases of the disease are polygenic and are caused by an interaction of environmental, lifestyle, and genetic factors [49]. As expected, a correlation has been found between obesity and a large number of genes determining fat and carbohydrate metabolism, eating behavior, circadian rhythms, and extracellular-matrix structure. The association of obesity with a number of innate-immunity genes has turned out to be interesting. In murine adipose perigonadal tissue, expression of 1304 transcripts correlates significantly with body weight; among the 100 most significantly correlating genes, 30% encode proteins that are characteristic of macrophages [29].

### 3.1. Obesity Caused by Knockouts (KOs) of Innate-Immunity Genes in Animal Models

Despite the difference in lipid metabolism and an incomplete match of fat depots between humans and rodents, model animals are widely used to assess the role of various innate-immunity genes in the development of obesity and low-grade inflammation in WAT. Experiments with mice have revealed that a KO of various innate-immunity genes makes animals either more or less susceptible to obesity either spontaneously or on a high-fat diet (HFD), as compared to controls. Additionally, a change in inflammatory status of WAT has been documented for some gene KOs. Table 1 shows the murine innate-immunity genes whose KO alters the adipose-tissue phenotype.

Among the genes whose knockout influences obesity in model animals are PRRs, cytokines, cytokine receptors, complement system genes, and some genes of proinflammatory signaling. Some of the obesity predisposition genes are common between mice and humans. On the contrary, mice differ significantly from humans, first of all, in life expectancy and in the organization of some genes and their regulation. The need to maintain a constant number of adipocytes through safe elimination of senescent cells makes the modulation of various processes inside human adipose tissue more complex. Experiments with KO mice are mainly based on the gene–environment (G × E) principle, in contrast to the bulk of studies on humans.

### 3.2. Human Innate-Immunity Gene Variants Associated with Obesity

Variants of innate-immunity genes for which a correlation with obesity has been demonstrated in humans are listed in Table 2.

#### 3.2.1. PRRs

*TLR* genes code for the main PRRs in host defense. Being anchored on the immune-cell plasma membrane (TLR 1, 2, 4–6, and 10) or endosomal membrane (TLR 3 and 7−9) in humans, they bind to components of the bacterial cell wall, pathogens’ DNA and RNA, and some endogenous molecules. TLRs launch common signaling pathways that cause transcription factors NF-κB and AP-1 to turn on genes expressing proinflammatory chemokines, cytokines, and cell adhesion molecules. In humans, all known TLRs are expressed in white adipocytes. Most TLR family members were more robustly expressed in the stromal vascular fraction (preadipocytes) than in the mature-adipocyte fraction [151]. Higher expression of TLR1, TLR2, TLR4, and TLR6 has been found in omental WAT in contrast to subcutaneous fat, regardless of metabolic status of the subject [11]. In mice, white adipocytes express TLRs 1–7 and TLR9 [152]. Aside from LPSs, resistin, free fatty acids, and mannose-binding lectin (MBL) can be ligands of TLR2 and TLR4 [153,154,155].

KOs of different *Tlr* genes affect mice differently. The absence of *Tlr2* alleviates insulin resistance and decreases levels of molecular markers of inflammation in obesity [50]. For *Tlr3* and *Tlr4* KOs, in addition to reduced inflammation, a protective effect against obesity is observed [51,52,53,156]. *Tlr5* data are contradictory: an HFD does not affect the weight gain in KO mice, but a low-fat diet yields different results [54,55]. *Tlr7* and *Tlr8* gene KOs protect mice against obesity, whereas *Tlr9* KO mice have higher weight [56,57]. The absence of the *Myd88* gene (encoding an adapter protein involved in signaling from all TLRs except TLR3) and of *Trif* (coding for the adapter protein involved in signaling from TLR3 and TLR4) suppresses adipose-tissue inflammation on an HFD but does not show an association with weight gain [75,156]. The list of TLRs in humans is different from that in mice: it lacks TLRs 11–13 and contains TLR10. Nonetheless, the functions of TLRs 1–9 as PRRs in mice and humans are the same. In contrast to mice, in humans, the association of the *TLR2* gene with obesity is documented, and missense variant rs5743708 G correlates with a higher risk of morbid obesity [92]. Opposite-acting variants have been identified in *TLR4* [93,94,95]. A stop gain variant in *TLR5* with minor allele frequency of ~5% protects against obesity [96].

CD14 is a coreceptor of TLR4 and TLR2 and participates in the detection of LPSs. It is highly expressed on monocytes, macrophages, DCs, and microglia and is present to a lesser extent in nonimmune cells. In obese people, CD14 is reported to be overexpressed significantly in monocytes, adipocytes, and the whole adipose tissue. A lipid mixture derived from dying cells directly binds to CD14 and promotes CD14 endocytosis via a mechanism similar to that of LPSs [157]. *Cd14* KO mice on a high-fat high-simple-carbohydrate diet had lower adiposity versus a control group [58]. Intron variant rs2569190 C of the *CD14* gene is associated with higher BMI in humans [98].

LRR-binding FLII-interacting protein 1 (LRRFIP1) is a cytosolic sensor of nucleic acids. The binding of LRRFIP1 to microbial nucleic acids triggers β-catenin and TLR signaling pathways and induces the production of type 1 interferon (IFN) and proinflammatory cytokines (through NF-κB activation). This protein performs multiple functions in the regulation of diverse biological systems and processes, but it is not clear how it can influence lipid metabolism [158]. Eight human *LRRFIP1* single-nucleotide polymorphisms (SNPs) correlate with a higher percentage of body fat, abdominal fat, and CRP level. The strongest evidence of association has been obtained for rs11680012 with abdominal adiposity phenotypes and rs11680012 with CRP levels. The C allele causes an arginine-to-threonine substitution in three of five LRRFIP1 protein isoforms; additionally, a splicing site may be altered. Rs11680012 is reported to be in linkage disequilibrium (LD) with noncoding transcript variant rs3806505. Carriers of the rare C allele of rs11680012 have 30% more abdominal adiposity than GG carriers do [97].

Mincle (macrophage inducible C-type lectin) is another PRR. This transmembrane protein is considered a danger receptor for both DAMPs and pathogen-associated molecular patterns. It launches host immunity against infection and induces inflammation during tissue damage. Mincle constitutively binds to a signal adaptor molecule: the Fc receptor γ chain. Human, but not murine, Mincle is mainly expressed on macrophages, monocytes, neutrophils, and DCs, and the expression is higher in the adipose tissue of obese mice and humans [159]. The function of this receptor appears to be complex. It is induced by LPSs, trehalose-6,6′-dimycolate (a mycobacterial cell wall component), and fungi. Mincle functions as a direct receptor of cholesterol crystals, sitosterol, and desmosterol. It is localized in crownlike structures of WAT and is thought to be a sensor of cell death. It triggers NF-κB signal transduction followed by the synthesis of proinflammatory cytokines and chemokines. Mincle can suppress TLR4 signaling and inhibits the apoptosis of neutrophils and macrophages [160,161]. There is no significant difference in body weight between Mincle-deficient (*Clec4e* KO) and wild-type mice on an HFD, whereas epididymal fat weight is significantly higher in Mincle-deficient mice [61]. A KO of the *Fcer1g* gene, encoding the Fc receptor γ chain (determining signal transmission from Mincle) diminishes diet-induced obesity and WAT inflammation [62]. Among humans, no association of the *CLEC4E* gene with obesity has been found.

NLRs are cytosolic PRRs. Most of them have immune functions: some of them control antigen presentation, others participate in pathogen or damage sensing, and a third group suppresses or modulates inflammation. The most thoroughly characterized NLRs are proteins that initiate the innate immune response to microbial molecules or DAMPs. They form inflammasome multiprotein complexes that are responsible for the activation of IL-1β, IL-18, and pyroptosis effector gasdermin through the cleavage of proteins pro-IL-1β, pro-IL-18, and GSDMD by caspase 1, 4, or 5. [162]. NOD-like receptor family pyrin domain-containing 1 (NLRP1) has been shown to be expressed in lymphocytes and monocytes/macrophages and to be triggered by altered homeostasis [163]. Unlike in humans, the genome of mice contains three *Nlrp1* paralogs (*Nlrp1a, Nlrp1b*, and *Nlrp1c*), which have different expression profiles [164]. Additionally, human NLRP1 has an N-terminal pyrin domain, which is absent in rodent NLRP1 proteins [163]. *Nlrp1a^−/−^Nlrp1b^−/−^Nlrp1c^−/−^* mice are reported to develop obesity spontaneously [64]. In humans, other *NLR* genes are associated with obesity. The inflammasome complex formed by NOD-like receptor protein 3 (NLRP3) functions as an upstream trigger of NF-κB signaling via activation of caspase 1, which in turn processes the cytosolic precursors of IL-1β, IL-18, and gasdermin. The NLRP3 inflammasome can act as a metabolic danger sensor toward a wide range of molecular patterns including cholesterol crystals, oxidized low-density lipoprotein cholesterol, and palmitate [165]. A group of inhibitory NLRs attenuates signaling cascades initiated by other families of PRRs. All four known regulatory NLRs have been proven to inhibit NF-κB signaling. It is assumed that these NLRs do not form inflammasomes but rather some other multiprotein complexes. One of the regulatory NLRs, NLR family CARD domain-containing 3 (NLRC3), inhibits TLR-dependent activation of the transcription factor NF-κB through interaction with adaptor protein TRAF6. Moreover, it has been revealed that NLRC3 can suppress IFN responses via interaction with STING (intracellular DNA-sensing protein that induces downstream IFN responses) [162,166]. *NLRP3* rs10754558 G in the 3′ untranslated region (UTR) protects humans from obesity [101]. Several research articles on different populations have revealed a correlation of *NLRC3* noncoding transcript variant rs758747 T with obesity [95,99,100]. A KO of the *Casp1* gene leads to obesity in an age- and sex-dependent manner in mice [63]. By contrast, effector caspases and/or adaptor molecules of the inflammasome are virtually monomorphic in humans [163].

FFAR2 and FFAR4 are expressed on the surface of macrophages and adipocytes. In adipose tissue, they play a dual immunometabolic role: they control the differentiation of white adipocytes, induce adipocyte “browning”, and modulate an immune response. FFAR4 binding to ligands has been shown to inhibit proinflammatory NF-κB and JNK signaling pathways, thus reducing macrophage-mediated inflammation in WAT. FFAR2 stimulates TNF secretion by M2 (but not M1) macrophages [167]. In knockout animals on an HFD, opposite effects of these FFARs have been registered: the absence of the *Ffar4* gene enhances obesity and inflammation, whereas the absence of the *Ffar2* gene diminishes weight and suppresses the migration of macrophages in mice [59,60]. Missense variant rs116454156 A of the *FFAR4* gene is associated with a higher risk of obesity [102].

#### 3.2.2. NF-κB Signaling

The main PRR-activated signaling cascade is the NF-κB pathway. It regulates both innate and adaptive immunity. NF-κB–dependent gene expression is required for immune-system development, pathogen recognition, inflammation, and cell survival during an immune response [168]. The NF-κB family of polypeptides consists of five members: NF-κB1 (p50), NF-κB2 (p52), RelA, RelB, and Rel, which form homo- or heterodimers complexed with member of the IκB family of inhibitor proteins in the cytosol. One of the most thermodynamically stable NF-κB dimers is NF-κB1–RelA; it is present at substantial levels in most cell types. Known inducers of NF-κB activity are highly diverse and include reactive oxygen species, TNF, IL-1β, and LPSs. These extracellular signals activate the IκB kinase, which in turn phosphorylates the IκB protein in a cytosol complex. As a consequence, the inhibitor protein dissociates from the NF-κB dimer, and the latter rapidly relocates into the nucleus, where it can bind to specific DNA sequences [168]. There are three classic IκB proteins: IκBα, IκBβ, and IκBε. Functions of IκBα and IκBε are more complicated; the induction of NF-κB activity upregulates IκBα and IκBε, and these newly synthesized proteins can enter the nucleus on their own to competitively remove NF-κB from DNA and return it to the cytoplasm [168]. It has been revealed that activation of NF-κB attenuates the programmed cell death induced by TNF receptors and several other stimuli [169].

A KO of the *Nfkb1* gene lowers adiposity [85]. An *Ikbke* KO prevents obesity and inflammation in mice on an HFD [87,88]. Being stimulated by obesity and an HFD, mediobasal-hypothalamus astrocytes can participate in central nervous system (CNS) immune responses and modulate feeding behavior. An astrocyte-specific deletion of the *Ikbkb* gene protects mice from diet-induced obesity [86]. As in mice, NF-κB signaling has been implicated in obesity in humans. 2KB upstream variant rs28362491 (ins/ins genotype and ins allele of 94 ins/del ATTG polymorphism) of the *NFKB1* gene predisposes to morbid obesity [92]. In a G × E study, it was shown that the intron rs3747811 TT genotype of *IKBKB* (inhibitor of nuclear factor kappa B kinase subunit β) affects the BMI and waist circumference depending on some *SOCS3* gene SNPs [150]. Perhaps the observed effect of NF-κB on WAT is related to its role in the CNS [86].

#### 3.2.3. The Complement System

This system is the first line of antimicrobial defense. The main consequences of its activation are phagocytosis of foreign material and damaged or altered host cells, recruitment of additional phagocytes, and the emergence of the cell-killing membrane attack complex (lytic pore in the membrane of a cell). The complement system cascade can be initiated via one of three pathways: a classic one (through sensing of IgM, IgG, and CRP), an alternative, i.e., spontaneous one, and a lectin pathway (through sensing of MBL and ficolins). It consists of small proteins circulating in the blood as inactive precursors, which, when activated, undergo sequential cleavage steps and interact with other proteins and specific receptors. During proteolytic events, biologically active protein fragments originally termed anaphylatoxins are generated: C3a and C5a from all three pathways (and outside the complement cascade) and C4a primarily from the classic pathway. These protein fragments are rapidly metabolized by carboxypeptidases, forming des-arginated (desArg) fragments [170]. C3a and C4a have been suggested to be both antibacterial agents and adaptive-immunity and metabolic regulators. C5a, C4a, and C3a are involved in neural development and organ regeneration. Three receptors of anaphylatoxins and their desArg forms have been found. In humans, C3aR is a receptor of C3a, and C5a1R binds both C5a and C5a desArg, whereas C5a2R is a possible receptor of C3a desArg [170,171]. All three receptors are expressed in macrophages, adipocytes, and preadipocytes to various degrees [172].

MBL recognizes and binds to mannose and N-acetylglucosamine on the surface of many microorganisms, including bacteria, yeast, and some viruses. This binding launches the lectin complement cascade, enhancing opsonophagocytosis of pathogens and cellular debris. Additionally, MBL can directly interact with macrophage receptors and alter cytokine expression [155,171,173]. In contrast to humans, mice have two active MBL-encoding genes. *Mbl1* + *Mbl2* KO mice display increased adipocyte size and greater influx of macrophages into adipose tissue [84].

Acute-phase protein CRP is synthesized by hepatocytes and released into the circulation in response to inflammatory stimuli, including TNF and IL-6, synthesized by WAT macrophages and adipocytes in obesity. CRP has native pentameric and monomeric conformations. Proinflammatory monomeric CRP is formed after the dissociation of the membrane-bound pentamer [174]. CRP can bind to phosphocholine, polysaccharides, a polycationic protein, and cholesterol on the membranes of bacteria and damaged cells. This event results in subunit rotation facilitating the interaction of CRP with complement component C1q, triggering the destruction of these cells by opsonin-mediated phagocytosis. The monomeric form interacts with receptors FcαR and FcγR of monocytes to initiate proinflammatory signaling [174].

Experiments on rats indicate that a *Crp* KO causes both a reduced weight gain on an HFD and smaller food intake. The latter is apparently induced by the recently discovered direct control of leptin signaling by CRP [79]. Carriage of the intron rs1130864 AA genotype and ACCC haplotype for rs1130864 (intron), rs1205 (3′ UTR), rs2794521 (2KB upstream), and rs3093062 (2KB upstream) of the *CRP* gene is associated with a higher BMI in Mexicans [138]. Complement component C3 plays a central part in the activation of the complement system. It is required for all complement activation pathways. C3 is produced primarily by hepatocytes but also by adipocytes, macrophages, and endothelial cells. Plasma C3 concentration correlates with the BMI, fat distribution, metabolic syndrome, and diabetes mellitus. It has been revealed that C3 concentration may increase by 50% postprandially [139,172]. The respective preproprotein is proteolytically processed to generate subunits C3a and C3b. In mice, a *C3* KO and *C3ar* KO attenuate the weight gain on an HFD [80,81]. *C5ar1* KO mice have greater adiposity and larger adipocyte size but smaller accumulation of total and proinflammatory M1 macrophages and increased expression of anti-inflammatory IL-10 [82]. While on an HFD, *C5ar2* KO mice feature smaller adipocyte size and a lower triglyceride level than do control animals [83]. Missense variant rs2230199 G in the human *C3* gene correlates with a higher BMI in Europeans [139]. It is assumed that the observed association of C3, C3a, and C5ar2 with lipid metabolism is explained by the fact that the metabolite C3a desArg described initially as acylation-stimulating protein (ASP) acts as an adipocyte-derived hormone, increasing diacylglycerol acyltransferase activity and glucose transport and stimulating lipid storage in adipose tissue [170]. Of note, complement factor D (adipsin) is a peptidase secreted by adipocytes into the bloodstream. It is necessary for C3bBb convertase activation in the alternative complement pathway and as an adipokine simultaneously [171]. Thus, many components of the complement system have a dual function affecting both an innate immune response and metabolism [175].

#### 3.2.4. Interleukins and Their Receptors

Interleukins are cytokines synthesized by immune cells and used to exchange information between cells and fine-tune various complex processes, including the development and completion of inflammation. Their functions depend on the cell type and can be redundant. ILs are conventionally categorized into proinflammatory and anti-inflammatory proteins according to the signaling cascades that they can launch by binding to cognate receptors. In adipose tissue, proinflammatory cytokines normally regulate extracellular-matrix remodeling, neovascularization, and the mobilization of fat stores and thermogenesis, in particular, they take part in the process of weight loss in response to physical exercise. Several interleukins—IL-6, IL-7, IL-8, IL-10, and IL-15—are released from skeletal muscle following physical activity [176,177]. Anti-inflammatory ILs prevent the acute inflammatory response caused by the activation of immune cells in adipose tissue. The interleukin 1 family includes proinflammatory IL-1α, IL-1β, IL-18, IL-33, IFNγ, and other ligands and antagonistic receptors. IL-1α, a proinflammatory acute-phase cytokine, can be expressed in adipose tissue by fibroblasts, macrophages, DCs, and T cells. Its downstream cascade, just as IL-1β signal transduction, includes IL-1R1, IL1RAP, MyD88, and IRAK4 and leads to the launch of proinflammatory NF-κB and MAPK signaling pathways [178]. Mice with this gene knocked out exhibit reduced adiposity [65]. As in mice, a correlation of *IL* genes with obesity has been shown in humans. Two variants found in *IL1A* (missense rs17561 A and 2KB upstream rs1800587 A) are associated with a higher BMI both together and separately in different populations [103,104].

Macrophages and monocytes are major IL-1β–producing cells. IL-1β shares a signaling cascade with IL-1α but needs to be proteolytically processed into its active form. The inflammasome activation that is mediated by cytoplasmic PRRs supplies caspase 1 for this processing. As a result of pyroptosis, active proteins IL-1β and IL-18 are released into the extracellular space and induce inflammation [163]. In addition, it is believed that pro-IL-1β can be processed by microbial proteases independently of inflammasomes [179]. *Il1b* KO mice have larger fat depots with a similar degree of adipocyte hypertrophy (as a result of hyperplasia) and enhanced M1 polarization [66]. Different genotypes of rs1143627 (in *IL1B)* have distinct phenotype correlations; A/G heterozygotes among elderly Swedish men have less total fat and a lower BMI, whereas AA genotype carriers in Japan are at a higher risk of obesity [105,106]. Synonymous variant rs1143634 A predisposes Swedish men to lower total fat mass and BMI [107].

IL-1 receptor antagonist (IL-1RN) is an anti-inflammatory cytokine and participates in the pathogenesis of both acute and chronic inflammatory diseases by binding to the same IL-1 receptors as IL-1α and IL-1β do. IL-1RN is strongly secreted by WAT. In the serum of obese patients, the soluble IL-1RN level correlates with the BMI [180]. A KO of the *Il1rn* gene causes obesity resistance in mice [74]. Just as its murine counterpart, human *IL1RN* is reported to be associated with obesity, and variant rs4252041 C in the 3′ UTR and synonymous rs419598 C predispose Swedish men to higher total fat mass and body weight [108]. *IL1RN* intronic variable number tandem repeat (VNTR) polymorphism rs2234663 (allele 2 of an 86 bp repeat) is in LD with rs419598 and correlates with greater total body fat and higher overall adiposity in different populations [107,109].

A KO of the IL1 receptor type 1 gene (*Il1r1*), which codes for a receptor of IL-1α, IL-1β, and IL-1RN, causes maturity onset obesity on a normal diet but reduces local adipose-tissue inflammation on an HFD despite almost unchanged immune-cell recruitment [72,73]. It has been theorized that the observed association of *IL1* cytokine family genes with obesity in mice may be due to leptin dysregulation and consequent hyperphagia rather than direct modulation of adipocyte function because *Il1r1* and *Il6* KO mice develop leptin resistance [181]. Interleukin 1 receptor accessory protein (IL1RAP) is a component of the IL-1 receptor complex, which initiates signaling events that result in the expression of IL-1–responsive genes. IL1RAP expression is present in human adipocytes and preadipocytes. Two *IL1RAP* variants—rs9990107 A in a 2KB upstream region and rs3836449 delCAGGGTGCCCCT in an intron—are associated with a higher obesity risk in a Korean population [128].

IL-4 is a proinflammatory cytokine secreted by activated T helper 2 lymphocytes and several types of innate-immunity cells, including mast cells, basophils, and eosinophils; it executes many biological tasks in adaptive and innate immune systems. As part of innate immunity, IL-4 is sufficient for antihelminth defense. In experiments on mice, eosinophils are reported to be the main source of IL-4 in adipose tissue [182]. In humans, IL-4 is able to bind to two types of receptor, differing in one of the subunits: the type I receptor binds exclusively to IL-4, whereas the type II receptor binds to both IL-4 and IL-13. These ILs promote the proliferation, survival, adhesion, and chemotaxis of mast cells [183]. IL-4 and IL-13 promote the differentiation of alternatively activated macrophages in brown adipose tissue during hypothermia. Additionally, IL-4 acts directly on adipose tissue and facilitates lipolysis [184]. It is possible that the correlations of IL-4 with adiposity are due to its participation in thermoregulation. *IL4* contains a 70-bp VNTR polymorphism within intron 3 (rs2234665), and two common alleles are B1 and B2, which have two and three tandem repeats, respectively. This locus may affect mRNA splicing, thereby giving rise to different splice variants. The B2 allele is associated with a higher total body fat amount and greater overall adiposity [109].

IL-6 is an important mediator of fevers and of the acute phase response. It seems to be overproduced in WAT during obesity. IL-6 is secreted by macrophages in response to pathogen-associated molecular patterns and stimulates acute-phase protein synthesis. It possesses a dual function as a proinflammatory cytokine and anti-inflammatory myokine. Plasma IL-6 levels can rise up to 100-fold in response to physical exercise. Muscular IL-6 regulates metabolism rather than inflammation [177]. *Il6* KO mice are characterized by maturity onset obesity, underexpression of lipolysis- and thermogenesis-related genes, and the absence of anti-obesity effects of exercise training [67,68]. Several studies have evaluated the correlation of *IL6* gene variants with indicators of obesity in humans, and the obtained data are contradictory. For instance, it has been demonstrated that the rs2069845 G allele predisposes to obesity and greater weight, BMI, and waist and hip circumferences in Indian children. Intronic rs1800795 has proven to be in moderate LD with a common variant: rs2069845 [110]. Findings about the latter SNP are inconsistent [111,112,185]. Intron variant rs1800796 C is associated with greater waist circumference in Mexican-Americans [113]. White men carrying *IL6* 3′ near-gene variant rs10242595 A have lower fat mass [114].

IL-6 receptor is a protein complex (IL6ST–GP130) consisting of the IL6R protein and IL-6 signal transducer, a receptor subunit also shared by many other cytokines. The *IL6R* gene encodes a subunit of the IL6 receptor complex. There are membrane-bound and soluble forms of IL-6R, and both can bind to IL-6. The soluble form’s complex can then be assembled into a complex with GP130 as well. The homodimerization of the receptor complex can trigger JAK–MAPK, JAK–STAT3, PI3K–AKT, and SRC-YAP pathways, which modulate proliferation, recruitment, survival, and growth of cells. JAK–MAPK signaling also takes part in acute-phase protein production [186]. Missense variant rs2228145 A of *IL6R* correlates with greater abdominal obesity and a higher BMI in various populations. It is known to be in LD with rs71586016 [115,116,117]. The latter is a microsatellite repeat polymorphism whose common intronic 149-bp allele may predispose to a higher BMI [118]. Allele rs7514452 C in the 3′ UTR of *IL6R* predisposes Indian children to obesity [110]. Mice with a hepatocyte-specific *Il6r* conditional KO have no alterations in body weight and fat content [187].

IL-10 is a major anti-inflammatory cytokine. It has strong suppressive effects on the inflammatory host response mediated by macrophages and lymphocytes and potently inhibits the production of proinflammatory cytokines. Low IL-10 circulating levels are reported to be associated with obesity and metabolic syndrome [28]. In Italian whites, the *IL10* rs1800872 TT genotype separately and within promotor haplotype “rs1800896, rs1800871, and rs1800872 (TAT/TAT genotype)” predisposes to obesity. The risk haplotype correlates with lower transcriptional activity of *IL10* [119].

IL-15 belongs to the IL-2 cytokine superfamily and is expressed by monocytes, macrophages, and muscle cells. It regulates a variety of processes in both innate and adaptive immunity. IL-15 exerts pro- and anti-inflammatory actions; its levels are detectable in the circulation both in humans and mice [188]. IL-15 is believed to be a myokine, and there is a rapid increase in circulating levels of IL-15 in response to exercise [176]. There are conflicting reports regarding whether IL-15 is expressed by adipocytes. In mice, a KO of the *Il15* gene results in a significantly greater weight gain without altering appetite, whereas IL-15 treatment results in significant weight loss in *Il15* KO mice and mice with diet-induced obesity independently from food intake [69]. IL-15 and IL-15 receptor subunits are expressed in several regions of the CNS, and they are assumed to be involved in hypothalamic pathways of energy homeostasis [189]. As in mice, *IL15* is reported to be associated with obesity in humans, and negative correlations between circulating IL-15 levels and both total and abdominal fat have been demonstrated [188]. Variants intronic rs1589241 T, noncoding rs1057972, and downstream intergenic rs2099884 T predispose to a higher BMI [120,121].

IL-15 receptor α (IL15RA) codes for both membrane-bound and soluble forms that can modulate IL-15 secretion and bioactivity. The IL-2Rβ and IL-2Rγ subunits of IL-15R are responsible for signal transduction, while IL-15Rα primarily implements high-affinity binding of the heterotrimeric receptor complex to IL-15 [188]. *Il15rα* KO mice are leaner than controls and have higher body temperature. Common variant rs3136618 T in intron 5 of *IL15RA* predisposes to normal-weight obesity (a normal BMI but greater than 30% of body fat and the presence of several markers of metabolic syndrome) in white women [122]. Circadian alterations of locomotor and metabolic activity indicate a direct action of IL-15Rα in the hypothalamus [189]. It is possible that the effects of *IL15* and *IL15RA* variants on WAT are caused by the respective proteins’ functions in the CNS.

IL-17 is a proinflammatory cytokine expressed by innate-like T cells and macrophages [70,190]. It interacts with receptors of the IL-17R family, which initiate downstream inflammatory NF-κB and MAPK signaling cascades. IL-17 inhibits differentiation of mouse preadipocytes into adipocytes. *Il17* KO mice have lower weight on an HFD [70].

IL-18 is a proinflammatory cytokine that belongs to the IL-1 superfamily. It is produced by macrophages, keratinocytes, osteoblasts, intestinal epithelial cells, pituitary gland cells, and adrenocortical cells, suggesting that it has pleiotropic functions. IL-18 participates in host defense against various bacterial, protozoan, and helminthic infections through strong induction of IFNγ, nitric oxide, and reactive oxygen species in phagocytes. Similar to IL-1β, IL-18 is generated as a biologically inactive precursor, pro-IL-18, which needs to be processed by a protease. Liberation of IL-18 via the activation of NLRP3, NLRP1, MEFV, NLRC4, AIM2, NLRP6, and NLRP9b inflammasomes is well documented [163,191]. In addition to caspase 1, the cleavage of pro-IL-18 can be carried out by caspase 8, proteinase 3, and granzyme [191]. IL-18 receptor (IL-18R) consists of an inducible ligand-binding component—IL-18Rα (IL-18R1)—and a constitutively expressed signal-transducing component—IL-18Rβ (IL-18RAP). The IL-18Rβ chain is required for high-affinity binding and cell signaling. Cytoplasmic TIR domains of the receptor complex interact with MyD88, driving the activation of transcription factors NF-κB and AP-1. IL-18R is expressed by T cells, NK cells, basophils, mast cells, epithelial cells, and nerve cells and is involved not only in immune responses but also in cell survival and differentiation [191]. Mice partially (*Il18*^+/−^) or totally (*Il18^−^*^/−^) deficient in IL-18 are hyperphagic. Adult *Il18^−^*^/−^ mice gain 2- to 3-fold more weight than controls do per unit energy consumed of a low- or high-fat diet. The evidence suggests that endogenous IL-18 signaling modulates food intake [71]. KOs of *Il18* and *Il18r1* lead to spontaneous obesity [75]. Intracerebral ventricular administration of IL-18 diminishes food intake in a dose-dependent manner in HFD-fed wild-type mice [192]. Additionally, *Il18* KO mice feature impaired activation of brown adipocytes. At the same time, *Il18r1* KO mice are protected from an acute body temperature drop, thus displaying more brown-like structure of fat [193]. Obese humans have higher levels of serum IL-18 [194]. The *IL18* gene is expressed in human subcutaneous and visceral adipose tissue, both in mature adipocytes and in the stromal-vascular fraction. Expression of genes *IL18R1* and *IL18RAP* is also present, mirroring that of *IL18*. The *IL18* mRNA level can rise more than 900-fold in response to TNF in human adipocytes differentiated in culture [195]. Carriage of rs1946518 T at 2KB upstream of *IL18* has been implicated in obesity in various populations [123,124]. Intron variant rs3882891 G appears to protect against obesity according to a G × E study [125]. In humans, an association of *IL18RAP* (encoding a signal-transducing chain of IL-18R) with obesity has been documented. Variant rs7559479 G in the 3′ UTR in this gene is protective against obesity, whereas intron variant rs2293225 T and rs2293225 T in the 3′ UTR predispose to obesity [126]. It has been proposed that IL-18 is more likely to have its effect on the hypothalamus rather than on adipose tissue itself.

IL-33 is an IL-1 cytokine family member. It is expressed in epithelial and endothelial cells, adipocytes, preadipocytes, and macrophages; IL-33 is present as an active full-length chromatin-associated protein in the nucleus and is released when cells undergo necrosis or are stressed. Inflammatory proteases from neutrophils and mast cells can process full-length IL-33 into shorter, 10- to 30-fold more potent polypeptides. On the contrary, during apoptosis, the full-length protein is inactivated by cleavage by apoptotic caspases (caspases 3 and 7). IL-33 performs its functions via a heterodimeric receptor composed of IL-1 receptor–like 1 (IL1RL1, also known as ST2) and IL-1 receptor accessory protein (IL1RAP). Both receptors are widely expressed, particularly by innate-immunity cells and T helper 2 cells. This receptor complex activates such signaling proteins as MyD88, TIRAP, IRAK1, IRAK4, and TRAF6 to induce a release of IL-4, IL-5, IL-10, IL-13, and MCP-1, thereby exerting either pro- or anti-inflammatory effects (among them, underexpression of adipogenic and metabolic genes) [196,197]. In humans, intron variant rs7044343 T of *IL33* protects against central obesity [127]. Minor allele frequency of seven SNPs of the *IL1RL1* gene (rs3771180, rs13431828, rs3214363, rs3771175, rs10204137, rs4988958, and rs10206753) is higher in an obese human sample and minor allele frequency of four SNPs (rs1420101, rs12905, rs3821204, and rs12712142) is lower there [128]. Carriers of 2KB upstream variant rs9990107 A and intron variant rs3836449 delCAGGGTGCCCCT (in the *IL1RAP* gene) are at a higher obesity risk [128]. In mice, a KO of *Il1rl1* does not affect obesity levels on an HFD [198].

#### 3.2.5. Other Cytokines

Another difference in lipid metabolism between humans and mice is the additional function of resistin (RETN). Although murine resistin is expressed primarily by adipocytes, human resistin is predominantly expressed by peripheral blood mononuclear cells and macrophages. Resistin genes in rodents and humans have markedly divergent promoter regions. In humans, resistin is probably more responsible for immunity than for the regulation of glucose homeostasis [199]. Its expression in mouse adipocytes is stimulated by high glucose levels but is suppressed by TNF, whereas human resistin is induced by LPSs, TNF, IL-6, IL-1β, and resistin itself in monocytes and macrophages [200]. This research demonstrated that TLR4 serves as a receptor mediating the proinflammatory effects of resistin in human cells [153]. *RETN* 2KB upstream variant rs1862513 C has been found to correlate with a higher BMI [132,133]. This finding is in contrast to a study indicating that *Retn* KO mice do not manifest any alteration in adiposity [201].

IFNs are cytokines with antiviral, antitumor, and antiproliferative properties. Innate-immunity type I and type III IFNs provide local protection during virus entry into cells. In contrast, type II IFN (IFNγ) is primarily produced by immune cells (including NK cells and activated T cells) to mediate adaptive immunity. IFNs limit virus replication and propagation by changing the transcription of host genes responsible for various microbicidal mechanisms. In response to an IFN, cells restrict their own protein synthesis and modify their own lipid metabolism and membrane composition. Activation of the p53 protein by an IFN leads to apoptotic death of the infected cell [202,203]. IFNγ treatment weakens insulin-stimulated glucose uptake in human adipocytes, attenuates insulin sensitivity, and suppresses differentiation of preadipocytes into adipocytes. The antiadipogenic impact of IFNγ is mediated by JAK–STAT1 pathway stimulation [204]. Human IFNγ receptor is a multimer composed of two IFNγR1 chains and two IFNγR2 chains. The *IFNGR1* gene encodes the ligand-binding chain (α-chain) of interferon gamma receptor (IFNγR1). It is expressed in macrophages and monocytes [205]. Intron variant rs13201877 G (in the *IFNGR1* gene) is associated with obesity in European populations [95,129].

There is evidence for the involvement of viruses (adenoviridae, herpesviridae, phages, transmissible spongiform encephalopathy viruses, some flaviviradae and retroviridae) in the genesis of obesity. During chronic infection, viruses reprogram host metabolism toward more lipogenic and adipogenic status [206]. It is believed that IFNs may directly reprogram cellular lipid synthesis and transport, in addition to IFNs’ indirect influence through an impact on viral infections [202].

#### 3.2.6. The Tumor Necrosis Factor Superfamily

The most important functions of superfamilies of TNF ligands and receptors (TNFSF/TNFRSF) are related to the immune system. They generally have proinflammatory properties due to their stimulation of the NF-κB signaling pathway but can also trigger apoptosis and other types of cell death. Within this group (19 ligands and 29 receptors), there are complex regulatory relations; some of the ligands have common receptors, and some of the receptors share some ligands. Some TNFRSF proteins contain death domains (DDs), which allow them to interact with one of several DD-containing adaptor molecules, leading to the formation of a scaffolding complex that initiates apoptosis. Non–death receptors lack a DD and either launch non–death signaling cascades or serve as decoy receptors. They are implicated in cell survival, proliferation, and cytokine production. TNF and two of its receptors (TNFR1 and TNFR2) are the best-studied members of these superfamilies. TNF is a multifunctional proinflammatory cytokine-like protein expressed by adipocytes, preadipocytes, endothelial cells, smooth muscle cells, fibroblasts, leukocytes, and macrophages. It can participate in the modulation of immune function, differentiation, proliferation, apoptosis, and energy pathways. During obesity, an increase in adipocyte size correlates with a greater release of TNF. This protein shows associations with total body fat rather than just visceral obesity [5,21]. Although TNF is primarily in the form of a trimeric type II transmembrane protein, it can also exist in a soluble form that is created by the cleavage activity of a matrix metalloproteinase. Whether membrane-bound or soluble, TNF can bind to both TNFR1 and TNFR2. TNFR1 possesses the DD, whereas TNFR2 does not. TNFR1 is expressed ubiquitously, but TNFR2 is expressed only on immune, neuronal, and endothelial cells. TNFR1 engagement by TNF promotes cell death and inflammation, whereas TNF binding to TNFR2 improves cellular survival and tissue regeneration. Despite their divergent functions, TNFR1 and TNFR2 both activate the transcription factor NF-κB. The switching between cell life and death decisions depends on diverse intracellular signaling events and crosstalk among pathways. Lymphotoxin α (LTα) is another member of the TNFSF superfamily; it is usually secreted as a soluble homotrimeric or heterotrimeric cytokine (in combination with LTβ) by activated T cells, resting B cells, myeloid-lineage cells, and nonhematopoietic cells. Homotrimeric LTα binds to TNFR1/2 just as TNF does [207]. In mice, it has been shown that TNF can take part in bitter-taste recognition, and its receptors TNFR1 and TNFR2 are located on taste bud cells [208]. A double KO of genes *Tnfrsf1a* and *Tnfrsf1b* (coding for murine TNFR1 and TNFR2) makes animals more obese and increases macrophage infiltration, but these macrophages predominantly have the anti-inflammatory M2 phenotype [76]. 2KB upstream variants rs1800630 A and rs1799964 C of the *TNF* gene predispose Sian Indians to obesity [134]. Promotor variant rs1800629 is most widely studied, but the findings are contradictory even in meta-analyses [135,136,209,210,211]. It is assumed that the correlation of allele A of this SNP with obesity is race specific. *LTA* gene minor allele rs915654 A carriers have a higher BMI and greater waist circumference [136]. Two intergenic variants (rs4343329 G and rs7225344 G) located near the *TNFRSF13B* gene (encoding one of the TNF receptors binding APRIL and the BAFF ligand) as well as intron variant rs4985700 G seem to be associated with greater visceral fat mass in Europeans [137].

#### 3.2.7. Chemokines and Their Receptors

Chemokines are cytokines with chemotactic properties and execute their specific functions by binding to rhodopsin-like G protein–coupled receptors. Some chemokines are considered proinflammatory and serve to recruit leukocytes to a site of infection or injury via changes in the cytoskeleton and conformation of adhesion molecules. Classically, a chemokine gradient for leukocyte trafficking is maintained by the immobilization of chemokines on extracellular matrices through their interaction with glycosaminoglycans. Chemokine synthesis is mainly induced by ILs, TNF, LPSs, and viruses.

CCL2 (monocyte chemoattractant protein 1, MCP-1) is secreted by monocytes, macrophages, DCs, fibroblasts, adipocytes, and preadipocytes and binds with high affinity to receptor CCR2. This chemokine recruits monocytes, basophils, memory T cells, and DCs to sites of inflammation. MCP-1 is also expressed by neurons, astrocytes, and microglia. CCR2 and CCR4 are two cell surface receptors that bind MCP-1 [212]. Adipocytes from obese individuals are characterized by significantly higher mRNA expression of MCP-1 [213]. *Ccl2* KO mice are protected against obesity [77]. In *Ccr2* KO mice, partial protection from diet-induced obesity has been revealed [78]. Expression of CC chemokines involved in monocyte chemotaxis (CCL2, CCL3, CCL5, CCL7, CCL8, and CCL11) and their receptors (CCR1, CCR2, CCR3, and CCR5) is higher in the adipose tissue of obese patients [214]. In Mexicans, promotor variant rs1024611 G of the *CCL2* gene is associated with a lower BMI and body fat proportion, whereas missense variant rs17998649 A of the *CCR2* gene correlates with a higher BMI [140]. Like many cytokines, CCL17 is proinflammatory; it is produced by DCs, macrophages, and monocytes. It is activated by such cytokines as TNF, IL-4, and IL-13 and interacts with its receptor CCR4. CCL17 induces chemotaxis of T helper 2 cells, regulatory T cells, basophils, and macrophages and inhibits TLR2 and TLR4 [215]. CCL20 is an adipochemokine whose expression is modulated by an anatomic arrangement of adipose tissue and by obesity severity. Mature adipocytes stimulate the migration of leukocytes by releasing CCL20, the receptor of which (CCR6) is expressed in leukocytes. The expression of adipocyte CCL20 and the number of WAT leukocytes positively correlate with the BMI and are greater in visceral than subcutaneous adipocytes. Mature adipocytes excrete a larger amount of CCL20 in obese people than in lean ones [8]. CCL17 gene variant rs223828 T and CCL20 gene variant rs6749704 T have been implicated in obesity in Tatars [141]. CX3CR1 is the only receptor for fractalkine (CX3CL1). CX3CL1 is a special chemotactic factor existing in both membrane-bound and soluble forms. It is involved in the adhesion and migration of leukocytes to sites of inflammation. CX3CL1–CX3CR1 signal transduction is triggered by INF-γ, IL-1β, TNF, IL-10, and IL-6 and affects inflammatory processes by communicating with different inflammatory signaling pathways, such as JAK–STAT, MAPK, AKT, and NF-κB. CX3CR1 is expressed on NK cells, monocytes, T cells, B cells, neurons, microglia, and smooth muscle cells [216]. The homozygous state of missense variants rs3732378 (AA) and rs3732379 (TT) (in the *CX3CR1* gene) is reported to be associated with greater mean waist circumference in women, and rs3732378 (AA) with a higher obesity risk in the whole study population [142].

Macrophage migration inhibitory factor (MIF) is a pleiotropic inflammatory cytokine with chemokine-like functions and appears to be a regulator of innate immunity. MIF is stored in vesicle-like structures in the cytoplasm and is secreted in response to injury, LPSs, proinflammatory cytokines, hypoxia, and glucocorticoid hormones. It is produced by macrophages, monocytes, and epithelial, endothelial, mesenchymal, and anterior pituitary cells. Different assays suggest that MIF can exist as a monomer, dimer, or trimer [217,218]. Overexpression of *MIF* in adipocytes correlates with enlarged subcutaneous abdominal adipocytes, reduced circulatory adiponectin concentration, and impaired insulin action [219]. MIF binds the receptors CD74, CXCR2, CXCR4, and CXCR7. A MIF–CXCR2 interaction has been found to trigger the recruitment and arrest of rolling monocytes, thereby leading to their transendothelial migration. Furthermore, MIF indirectly enhances leukocyte arrest by inducing the expression of adhesion molecules or other chemokines. Upon receptor binding, several downstream signaling pathways are known to be launched in vivo, including ERK1/2, AMPK, and AKT cascades. In Turkey, the promotor rs755622 CC genotype of *MIF* predisposes to higher abdominal obesity [130]. Another promotor microsatellite repeat variant, rs5844572 (ins ATTC; 6, 7, and 8 VNTR) at position −794, correlates with a higher obesity risk in nondiabetic Japanese; in contrast, rs755622 is not associated with obesity in the same sample [131].

#### 3.2.8. JAK–STAT Signaling Components

The main signaling pathway triggered by ILs and IFNs is the JAK–STAT cascade; it modulates immunity, cell division, and cell death. Its downstream components are Janus kinases (JAKs) and signal transducer and activator of transcription proteins (STATs). After a cytokine binds to and induces the dimerization of the corresponding receptors, JAKs are coupled to and phosphorylate the receptors; STATs are phosphorylated by receptor-binding JAKs, are dimerized, and then are transported into the nucleus to regulate the transcription of target genes. The JAK family comprises four members: JAK1–3 and Tyk2, and the STAT family is composed of seven members, namely STATs 1–4, 5A, 5B, and 6. SOCSs are STAT-induced STAT-inhibitory proteins, and their expression is induced by active JAK–STAT signaling. There are eight members of the SOCS family: CIS and SOCS1–7. CIS and SOCS1–3 implement a negative feedback loop of cytokine signaling, and other SOCSs primarily control growth factor receptor signaling [220]. Additionally, modulation of NF-κB activity by SOCS1 and SOCS3 has been documented [221]. Proteins of the SOCS family can mark their targets for degradation by the ubiquitin pathway and inhibit the activity of enzymes by competing with substrates. SOCS1 can inhibit signals of IL-2, IL-4, IL-6, IL-7, IL-12, IL-15, IFNs, TNF, and LPSs, whereas SOCS2 inhibits IL-6 signals, and SOCS3 suppresses IL-2, IL-4, IL-6, IL-9, IL-11, and IFN signals [222]. Basal SOCS3 expression is reported to increase in obesity [223]. In mice, a correlation of several components of this signaling cascade with obesity has been found. Animals with an adipocyte-specific conditional KO of the *Jak2* gene are cold intolerant and susceptible to HFD-induced obesity [89]. Adipocyte-specific deletion of the *Stat5* gene promotes adiposity [90]. *Socs2* KO mice possess higher adipose tissue mass independently of diet [91]. Early lethality of *Socs1-* and *Socs3*-deficient mice has been revealed [222]. In humans, 2KB upstream variant rs4796793 C of the *STAT3* gene is associated with a higher BMI in European Americans [143], and the 3′ UTR rs1053005 CC genotype protects Han Chinese against obesity [144]. Intron variants rs8069645 G and rs744166 G, 3′ UTR rs1053005 C, and the missense rs2293152 CC genotype of *STAT3* correspond to a higher risk of abdominal obesity [145]. Promotor variants of the *SOCS1* gene—rs33977706 A and rs243330 T—respectively lower the BMI and increase obesity risk in Denmark [146]. 3′ UTR variant rs2280148 G of the *SOCS3* gene is associated with a higher BMI in different cohorts [147,148]. 3′ UTR rs7221341 C and rs4969168 A, 2KB upstream variant rs8070204 G, and intron rs4969170 G in the same gene predispose to a higher BMI [143,147,149]. According to a G × E study, carriage of intergenic rs6501199 G and rs4436839 G variants near the *SOCS3* gene predisposes whites to a higher BMI and Asians to greater waist circumference [150].

## 4. The Microbiota and Innate-Immunity Genes

Thus, genes of innate immunity may contribute to the obesity phenotype. For some of them, a dual function has already been reported: they participate in protecting the organism from invaders and regulate energy metabolism at WAT, muscle, brain, or whole-body levels. Of particular interest is the interaction of innate immunity with the gut microbiota. The immune system shapes the community of gut microorganisms into a configuration that is tolerated by the host and is beneficial for it via modulation of the secretion of mucin, antimicrobial peptides, and IgA [224]. The roles of TLRs, NLRs, and other PRRs have been demonstrated in the regulation of microbiota composition and protection from pathogenic bacteria, fungi, and viruses in the intestine [224,225]. The gut microbiome has been shown to differ between lean and obese people. Increased relative abundance of the Proteobacteria phylum of bacteria (in particular, Pseudomonadales) and decreased relative abundance of the Firmicutes phylum (in particular, *Oribacterium asaccharolyticum*, *Atopobium parvulum*, and *Fusobacterium nucleatum*) have been found in obese subjects compared with a control [226]. In different studies, lower relative abundance of Clostridiaceae and Ruminococcaceae families of bacteria in the Firmicutes phylum has been detected in obese children, as has greater relative abundance of the Proteobacteria phylum and the Coriobacteriaceae family in the Actinobacteria phylum [227]. The Actinobacteria phylum is less abundant and significantly less transcriptionally active in obese humans. Most bacterial species are more transcriptionally active in obesity-associated microbiotas [228]. The *Blautia* genus (Firmicutes) is over-represented, whereas *Christensenella minuta* (Firmicutes), *Akkermansia muciniphila* (Verrucomicrobia), and *Methanobrevibacter smithii* (the Archaea domain) are under-represented in obese persons. It has been suggested that some of the heritability of obesity might be explained by host gene defects that shift the composition of the gut microbiota toward microorganisms that can promote either obesity or leanness [229].

Numerous studies have been conducted to find a correlation between the host genotype and microbiota composition, which can potentially influence the predisposition to obesity. In mice, an “obese” microbiota has been found to enhance a capacity to extract energy from the diet. This trait is transmissible: colonization of germ-free mice with an “obese microbiota” results in a significantly greater increase in total body fat than does colonization with a “lean microbiota” [230]. The microbiota can promote fat storage in adipocytes through suppression of intestinal expression of a lipoprotein lipase inhibitor, Fiaf [231]. Genetic variants of several genes are associated with both obesity and relative abundance of certain bacteria in humans [228], but there is not enough information about innate-immunity genes. Nevertheless, in humans, these genes are reported to be associated with various alterations of the microbiome. A PRR called nucleotide-binding oligomerization domain-containing protein 2 (NOD2) is primarily expressed in peripheral blood leukocytes and can recognize the bacterial molecules that possess a muramyl dipeptide moiety (a cell wall component in both Gram-positive and Gram-negative bacteria). It stimulates NF-κB signaling [232]. Frameshift rs2066847 and missense rs2066845 variants of the *NOD2* gene are thought to be associated with shifts in the amount of some taxa of Firmicutes relative to all bacteria in the human ileum [233]. Variants rs8056611 and rs2357792 downstream of the *NOD2* gene correlate with the profile of gut microbial species [234]. NOD1 is a PRR that recognizes molecules containing a d-glutamyl-meso-diaminopimelic acid moiety, including bacterial peptidoglycan. Stimulation of NOD1 enhances caspase-9–mediated apoptosis and activates the transcription factor NF-κB [232]. Intron SNPs in the *NOD1* gene (rs12669082, rs41524946, rs55689059, and rs55841603) are associated with a gut microbiome shift [234]. In the same study, other, additional correlations of the microbiome with innate-immunity genes were observed. Among these, we can cite rs3091315 and rs3091316 located between genes *CCL2* and *CCL7*, rs12141575 between genes *IL23R* and *IL12Rb2*, and rs1800871 located in the 2KB upstream region of the *IL10* gene. Of note, the latter SNP has been implicated in obesity [119]. Microbiome composition in inflammatory bowel disease correlates with rs6871626 near *IL12B* and intronic rs4246905 of the *TNFSF15*, a member of the tumor necrosis factor ligand superfamily [235]. Missense variants rs4833095 C of the *TLR1* gene and rs5744174 C of the *TLR5* gene, synonymous variant rs3804099 C of the *TLR2* gene, and 2KB upstream variant rs10004195 A of the *TLR10* gene raise the risk of *Helicobacter pylori* infection [236]. *MEVF* is an NLR gene coding for a PRR component of the inflammasome complex that can recognize host Rho-GTPase inactivation by various bacterial toxins [237]. It is associated with familial Mediterranean fever, which is an autoinflammatory disorder characterized by periodic attacks. When comparing symbiotic microbiotas between 19 patients with familial Mediterranean fever and eight healthy individuals depending on *MEFV* genotypes, researchers revealed that bacterial composition substantially deviates from the norm in patients experiencing an attack or remission. Missense variants rs28940580, rs61752717, rs28940579, and rs104895097 as well as synonymous rs224225 and rs224224 of the *MEVF* gene are believed to correlate with microbiome composition [238]. Analysis of the metatranscriptome of lean and obese people on a self-reported Mediterranean diet has uncovered differential expression of 40 genes, including low transcription of the *TNFRSF13B* gene, a member of the superfamily of TNF receptors [228]. An SNP near this gene has been found to be associated with obesity [137]. Nonetheless, results of microbiome genome-wide association studies have been partially reproduced for only a few innate-immunity genes, and host genetic factors explain a small proportion of the variance in the gut microbiome. There is a need for larger standardized study populations and more rigorous statistical approaches that will allow the detection of correlations having a small effect size [228,239]. The same problem arises when results of different host genetic studies are compared.

## 5. Conclusions

Different components of innate immunity regulate the life cycle of adipocytes and intensity of lipid metabolism. Moreover, host genetic factors can influence microbiome composition and indirectly affect nutrition harvesting in the gut. Associations of various genotypes of innate-immunity genes with predisposition to obesity are race and ethnogeography specific in humans. This phenomenon may be due both to differences in genetic backgrounds, diet, and environment and various criteria for inclusion in the study (BMI > 25 or > 30, only with obesity complications or without). G × E studies are still scarce. Animal models cannot fully replace humans in the investigation into fat metabolism owing to differences in the regulation of some immunity genes associated with obesity. Nonetheless, it is obvious that the dual immunometabolic function of the many genes mentioned indicates a tight interaction and mutual influence of innate immunity and lipid metabolism.

## Figures and Tables

**Table 1:** Knocked out innate-immunity genes and corresponding WAT phenotypes in model animals (mice, unless indicated otherwise).

Knocked out Gene	Phenotype of WAT	References
*Tlr2*	Protection against accumulation of macrophages	[50]
*Tlr3*	Protection against high-fat-diet–induced obesity Attenuated macrophage infiltration	[51]
*Tlr4*	Prevention of obesity Lower macrophage infiltration	[52]
*Tlr4*	Reduced adipose-tissue levels of inflammatory markers and macrophage content	[53]
*Tlr2* or *Tlr4* but not *Tlr5*	No weight loss in obese mice when fed a low-fat diet	[54]
*Tlr5*	Decreased weight reduction after low-fat diet	[55]
*Tlr7* or *Tlr7* + *Tlr8*	Protection against body weight gain	[56]
*Tlr9*	Higher weight and more body fat; dramatic increase in M1 macrophage number	[57]
*Cd14*	Lower adiposity	[58]
*Ffar2*	Lower body fat mass, lower macrophage content	[59]
*Ffar4*	Obesity, higher WAT inflammation	[60]
*Clec4e*	Epididymal fat weight is significantly higher, WAT inflammation and fibrosis are attenuated, number of crownlike structures is significantly diminished	[61]
*Fcer1g*	Attenuated diet-induced obesity and WAT inflammation	[62]
*Casp1*	Obesity with age- and sex-dependent differences	[63]
*Nlrp1*	Spontaneous obesity	[64]
*Il1a*	Reduced adiposity	[65]
*Il1b*	Larger fat depots with similar degree of adipocyte hypertrophy, higher levels of adipogenesis markers, enhanced M1 polarization	[66]
*Il6*	Absence of anti-obesity effects of exercise training	[67]
*Il6*	Maturity onset of obesity	[68]
*Il15*	Larger amounts of body fat	[69]
*Il17*	Lower weight and reduced WAT inflammation	[70]
*Il18*	Spontaneous obesity	[71]
*Il1rI*	Maturity onset of obesity	[72]
*Il1rI*	Reduced WAT inflammation	[73]
*Il1rn*	Obesity resistance	[74]
*Il18*	Spontaneous obesity	[75]
*Il18r1*	Spontaneous weight gain	
*Tnfrsf1a + Tnfrsf1b*	Higher obesity, increased macrophage infiltration, but with predominance of anti-inflammatory M2 macrophages	[76]
*Ccl2*	Protection against obesity	[77]
*Ccr2*	Partial protection against diet-induced obesity	[78]
*Crp (*Rattus norvegicus*)*	Reduction in weight gain and food intake	[79]
*C3*	Reduction in weight gain, but no reduction in WAT inflammation	[80]
*C3aR*	Transient resistance to diet-induced obesity	[81]
*C5ar1*	Increased WAT, larger adipocyte size, weaker accumulation of total and proinflammatory M1 macrophages	[82]
*C5ar2*	Hyperphagia (~60% increase in total food intake) yet maintained same body weight; on HFD, average adipocyte size is significantly reduced	[83]
*Mbl1 + Mbl2*	Increased adipocyte size, greater influx of macrophages	[84]
*Nfkb1*	Reduction in obesity	[85]
*Ikbkb* in astrocytes	Protection against obesity	[86]
*Ikbke*	Protection against obesity	[87]
*Ikbke*	Protection against obesity and inflammation in WAT	[88]
*Jak2* in adipocytes	Susceptibility to HFD-induced obesity	[89]
*Stat5* in adipocytes	Increased adiposity	[90]
*Socs2*	Higher adipose-tissue mass independently of diet	[91]

**Table 2:** Genetic variants of innate-immunity genes associated with obesity in humans. Risk allele designation is given in accordance with the dbSNP nomenclature.

Gene	SNP ID, Risk Allele, and Location	Phenotype	Population	References
PRR, coreceptors, and regulators				
*TLR2*	rs5743708 G, missense	Higher risk of morbid obesity	Turkey	[92]
*TLR4*	rs11536889 C, 3′ UTR	Protection against overweight	Argentina	[93]
*TLR4*	rs4986790 G + rs4986791 T, missense (cosegregated among Europeans)	Increased total body fat and visceral fat	whites	[94]
*TLR4*	rs1928295 T, intergenic	Higher BMI	Europeans	[95]
*TLR5*	rs5744168 A, stop gained	Protection against obesity, lower BMI	Saudi Arabia	[96]
*LRRFIP1*	rs11680012 C, missense and potentially splice site	Higher abdominal adiposity and higher levels of inflammation markers	Canada	[97]
*CD14*	rs2569190 C, intron	Higher BMI	Korea	[98]
*NLRC3*	rs758747 T, noncoding	Higher risk of obesity	Europeans	[95]
*NLRC3*	rs758747 T, noncoding	Higher BMI	Pakistan	[99]
*NLRC3*	rs758747 T, noncoding	Higher BMI	Pima Indians	[100]
*NLRP3*	rs10754558 G, 3′ UTR	Protection against obesity	Brazil	[101]
*FFAR4*	rs116454156 A, missense	Higher risk of obesity	Europeans	[102]
Cytokines and cytokine receptors				
*IL1A*	rs17561 A–rs1800587 A, missense: 2KB upstream	Increased BMI	Mexico, male adolescents	[103]
*IL1A*	rs1800587 A, missense	Increased BMI	Korea, healthy obese women	[104]
	rs17561 A, 2KB upstream			
*IL1B*	rs1143627 A/G heterozygotes, 5′ UTR	Lower BMI, total fat	Sweden, elderly men	[105]
*IL1B*	rs1143627 AA, 5′ UTR	Higher risk of obesity	Japan	[106]
*IL1B*	rs1143634 A, synonymous	Lower total fat mass and BMI	Sweden, men	[107]
*IL-1RN*	rs2234663, allele II (2 VNTRs of 86 bp carriers), intron	Increased total fat		
*IL-1RN*	rs4252041 C, 3′ UTR	Higher total fat mass, body weight, and BMI	Sweden, men	[108]
	rs419598 C, synonymous	Higher total fat mass and body weight		
*IL-1RN*	rs2234663, IL-1RN*2 86 bp repeat VNTR, intron	Increased total body fat, higher overall adiposity	Malaysia	[109]
*IL4*	rs2234665, 70 bp repeat VNTR allele B2, intron			
*IL6*	rs2069845 G, intron moderate linkage disequilibrium with rs1800795	Increased risk of obesity, higher weight, BMI, waist and hip circumferences	India, children	[110]
*IL6R*	rs7514452 C, 3′ UTR			
*IL6*	rs1800795 GG, intron	Increased risk of obesity	Greece, children	[111]
*IL6*	rs1800795 G, intron	Increased risk of obesity.	Meta-analysis	[112]
*IL6*	rs1800796 C, intron	Greater waist circumference	Mexican-Americans	[113]
*IL6*	rs10242595 A, 3′ near gene	Decreased fat mass	white men	[114]
*IL6R*	rs2228145 A, missense	Higher abdominal obesity	Japan, men	[115]
*IL6R*	rs2228145 A, missense; rs4845623 A, intron; rs2229238 T, 3′ UTR	Higher BMI	Pima Indians	[116]
*IL6R*	rs2228145 A, missense	Increased risk of obesity	Mediterranean whites	[117]
*IL6R*	rs71586016 common 149-bp allele of microsatellite repeat polymorphism, intron	Higher BMI	Spain, women	[118]
*IL-10*	rs1800872 TT, IL-10 2KB upstream/IL-19 intron	Increased risk of obesity	Italia	[119]
	rs1800896, rs1800871, rs1800872 TAT/TAT genotype, IL-10 2KB upstream/IL-19 intron	Increased risk of obesity		
*IL15*	rs2099884 T, intergenic	Higher BMI	USA, children	[120]
*IL15*	rs1589241 T, intron	Higher BMI	USA	[121]
	rs1057972 A, noncoding			
*IL15RA*	rs3136618 T, intron	Women with normal-weight obesity (De Lorenzo syndrome)	whites	[122]
*IL18*	rs1946518 T, 2KB upstream	Increased risk of obesity	Korea, women	[123]
*IL18*	rs1946518 TT, 2KB upstream	Elevated risk of obesity and overweight	Pakistan	[124].
*IL18*	rs3882891 G, intron	Protective against obesity depending on linoleic acid consumption	Germany	[125]
*IL18RAP*	rs7559479 G, 3′ UTR	higher obesity risk and BMI	Spain	[126]
	rs2293225 T, 3′ UTR	lower obesity risk and BMI		
	rs2293225 T, intron	Lower obesity risk		
*IL33*	rs7044343 T, intron	Protective against central obesity	Mexico	[127].
*IL1RL1*	rs3771180 T, intron; rs13431828 T, 5′ UTR; rs3214363 del T, intron; rs1420101 C, noncoding; rs12905 G, noncoding; rs3771175 A, noncoding; rs3821204 C, noncoding; rs12712142 C, noncoding; rs10204137 G, missense; rs4988958 C, synonymous; rs10206753 C, missense	Higher obesity risk	South Korea	[128]
*IL1RAP*	rs9990107 A, 2KB upstream; rs3836449 delCAGGGTGCCCCT, intron			
*IFNGR1*	rs13201877 G, intron	Higher obesity risk	Europeans	[95]
*IFNGR1*	rs13201877 G, intron	Higher obesity risk	Germany, children	[129]
*MIF*	rs755622 CC, 2KB upstream	Higher abdominal obesity risk, higher risk of new-onset diabetes	Turkey	[130]
*MIF*	rs5844572 ins ATTC 6 VNTR, 2KB upstream	Higher obesity risk	Japan	[131]
*RETN*	rs1862513 CC, 2KB upstream	Higher BMI	India	[132]
*RETN*	rs1862513 C, 2KB upstream	Higher BMI	Tunisia	[133]
TNF superfamily				
*TNF*	rs1800630 A, 2KB upstream; rs1799964 C, 2KB upstream	Higher obesity risk	Asian Indians, adolescents	[134].
*TNF*	rs1800629 A, 2KB upstream	Higher BMI	Egypt	[135].
*TNF*	rs1800629 GG, 2KB upstream	Higher risk of abdominal obesity	French	[136]
*LTA*	rs915654 A, intron	Higher BMI, greater waist circumference		
*TNFRSF13B*	rs4343329 G, intergenic; rs7225344 G, intergenic; rs4985700 G, intron	Visceral fat mass	Europeans	[137]
Complement system				
*CRP*	rs1130864 AA, intron	Increased BMI	Mexico	[138]
	rs1130864 (Intron), rs1205 (3′ UTR), rs2794521 (2KB upstream), rs3093062 (2KB upstream) ACCC haplotype			
*C3*	rs2230199 G, missense	Higher BMI	Europeans	[139]
Innate-immunity chemokine and chemokine receptors				
*CCL2*	rs1024611 A, noncoding	Lower BMI and body fat proportion	Mexico	[140]
*CCR2*	rs17998649 A, missense	Higher BMI		
*CCL20*	rs6749704, T 2KB upstream	Obesity among patients with type 2 diabetes mellitus	Tatars	[141]
*CCL17*	rs223828 T, intron			
*CX3CR1*	rs3732378 AA, missense	Higher obesity risk	Canada	[142]
	rs3732379 TT, missense	Greater mean waist circumference in women		
NF-κB and JAK/STAT signaling components				
*NFKB1*	rs28362491 ins/ins genotype and ins allele 94 ins/del ATTG, 2KB upstream	Morbid obesity	Turkey	[92]
*SOCS3*	rs4969170 G, intron	Higher BMI	European Americans	[143]
*STAT3*	rs4796793 C, 2KB upstream			
*STAT3*	rs1053005 CC, 3′ UTR	Lower risks of both general obesity and central obesity	Chinese Han	[144]
*STAT3*	rs8069645 G, intron; rs744166 G, intron; rs1053005 C, 3′ UTR; rs2293152 CC, missense	Increased risk of abdominal obesity	Ireland	[145]
SOCS1	rs33977706 A, 2KB upstream	Lower BMI	Denmark	[146]
	rs243330 T, 2KB upstream	Higher obesity risk		
*SOCS3*	rs8070204 G, 2KB upstream; rs7221341 C, 3′ UTR; rs2280148 G, 3′ UTR	Higher BMI	Hispanic Americans	[147]
*SOCS3*	rs2280148 G, 3′ UTR	Higher BMI	Turkish, children	[148]
*SOCS3*	rs4969168 A, 3′ UTR	Higher weight	Han Chinese	[149]
*SOCS3*	rs6501199 G, intergenic	Higher BMI	whites	[150]
	rs4436839 G, intergenic	Greater waist circumference	Asians	
*IKBKB*	rs3747811 TT, intron	Higher BMI	whites	
